# Sedoheptulose-1,7-bisphospate Accumulation and Metabolic Anomalies in Hepatoma Cells Exposed to Oxidative Stress

**DOI:** 10.1155/2019/5913635

**Published:** 2019-01-13

**Authors:** Mei-Ling Cheng, Jui-Fen Lin, Cheng-Yu Huang, Guan-Jie Li, Lu-Min Shih, Daniel Tsun-Yee Chiu, Hung-Yao Ho

**Affiliations:** ^1^Department of Biomedical Sciences, College of Medicine, Chang Gung University, Taoyuan, Taiwan; ^2^Metabolomics Core Laboratory, Healthy Aging Research Center, Chang Gung University, Taoyuan, Taiwan; ^3^Clinical Metabolomics Core Laboratory, Chang Gung Memorial Hospital at Linkou, Taoyuan, Taiwan; ^4^Department of Medical Biotechnology and Laboratory Science, College of Medicine, Chang Gung University, Taoyuan, Taiwan

## Abstract

We have previously shown that GSH depletion alters global metabolism of cells. In the present study, we applied a metabolomic approach for studying the early changes in metabolism in hydrogen peroxide- (H_2_O_2_-) treated hepatoma cells which were destined to die. Levels of fructose 1,6-bisphosphate and an unusual metabolite, sedoheptulose 1,7-bisphosphate (S-1,7-BP), were elevated in hepatoma Hep G2 cells. Deficiency in G6PD activity significantly reduced S-1,7-BP formation, suggesting that S-1,7-BP is formed in the pentose phosphate pathway as a response to oxidative stress. Additionally, H_2_O_2_ treatment significantly increased the level of nicotinamide adenine dinucleotide phosphate (NADP^+^) and reduced the levels of ATP and NAD^+^. Severe depletion of ATP and NAD^+^ in H_2_O_2_-treated Hep G2 cells was associated with cell death. Inhibition of PARP-mediated NAD^+^ depletion partially protected cells from death. Comparison of metabolite profiles of G6PD-deficient cells and their normal counterparts revealed that changes in GSH and GSSG per se do not cause cell death. These findings suggest that the failure of hepatoma cells to maintain energy metabolism in the midst of oxidative stress may cause cell death.

## 1. Introduction

Reactive oxygen species (ROS) are implicated in a number of physiological and pathophysiological processes. Depending on their level, ROS can serve as signaling molecules to promote cell proliferation or as mediator of cell death. Exposure to relatively high levels of oxidant induces apoptosis and necrosis. ROS inflict damages to cellular macromolecules, which, if not repaired, elicit apoptosis and necrosis [1, 2]. Normally, cells are equipped with an arsenal of antioxidants to impose a control on ROS generation [3, 4]. For instance, glutathione (GSH) acts as substrate for antioxidative enzymes. Glutathione peroxidase catalyzes reduction of hydroperoxides, accompanied by oxidation of GSH to its disulfide form. The latter is reduced back to GSH through the activity of glutathione reductase. NADPH is needed as a coenzyme in the latter reaction. Inefficient NADPH production and GSH regeneration are known to promote death of cells under oxidative stress [5].

Maintenance of antioxidative defense depends on active metabolism. Oxidative stress rapidly increases the flux of glucose into the pentose phosphate pathway (PPP) and NADPH production. PPP activation is involved in cytoprotection against oxidative damage [6]. Consistent with this, glucose 6-phosphate dehydrogenase- (G6PD-) deficient cells are more susceptible to diamide-induced GSH depletion [5] and utilize different biochemical pathways in an attempt to maintain their GSH and NADPH pools [7, 8]. The GSH biosynthesis and NAD phosphorylation are upregulated at the cost of excessive energy usage. Moreover, the metabolic responses of erythrocytes to diamide differ from those of nucleated cells [7]. The interplay between oxidative stress, the antioxidant system, and metabolism is more complicated than what has been previously thought. It is interesting to study if other oxidants, such as H_2_O_2_, elicit metabolic responses unique from those of diamide treatment. Thorough understanding of metabolic changes in response to oxidants necessitates the application of metabolomics.

Intracellular NADPH/NADP^+^ and NADH/NAD^+^ are involved in maintenance of antioxidant defense and energy metabolism, respectively. Additionally, these pyridine nucleotides act as coenzymes in metabolism and have regulatory functions [9]. NAD^+^ is a precursor of cyclic ADP ribose [10] as well as a substrate for ADP ribosylation by poly-ADP-ribose polymerases (PARPs) [11], which are involved in processes such as DNA repair and cell death. The heightened PARP activation may activate programed cell death despite antioxidant replenishment [12].

In this study, we used a LC-MS-based metabolomic research platform [8, 13] for studying the early changes in metabolite profile accompanying H_2_O_2_-induced death. Our findings indicate that PPP is activated and production of S-1,7-BP, an unusual PPP intermediate, increases in H_2_O_2_-treated cells. PPP and the NAD kinase (NADK) pathway are activated to furnish sufficient reducing equivalents. ATP and NAD^+^ pools dwindle, leading to dysfunction in metabolism. Inhibition of PARP-mediated exhaustion partially protects cells from death.

## 2. Material and Methods

### 2.1. Reagents

Unless otherwise stated, all chemicals were obtained from Sigma-Aldrich (St. Louis, MO, USA). Dulbecco's modified Eagle's medium (DMEM), fetal calf serum (FCS), penicillin, streptomycin, amphotericin, and trypan blue were purchased from Thermo Fisher Scientific Inc. (Waltham, MA, USA). Anti-G6PD antibody was purchased from Genesis Biotech (Taiwan); mouse anti-NADK monoclonal antibody (sc-100347) was available from Santa Cruz Biotechnology (CA, USA); rabbit anti-phospho-AMPK*α* (Thr172) (40H9) and rabbit anti-AMPK*α* (D5A2) antibodies were obtained from Cell Signaling Technology (Danvers, MA, USA). Anti-actin antibody and anti-rabbit and anti-mouse IgG antibodies were available from Sigma-Aldrich (St. Louis, MO, USA). Western Lightning Chemiluminescence Reagent Plus was purchased from PerkinElmer (Waltham, MA, USA).

### 2.2. Cell Culture and Cell Viability Determination

Hep G2 cells were cultured in DMEM supplemented with 10% FCS, 100 units/ml penicillin, 100 *μ*g/ml streptomycin, and 0.25 *μ*g/ml amphotericin at 37°C in a humidified atmosphere of 5% CO_2_ as previously described [5].

Cell viability was determined using the neutral red uptake assay [5]. After H_2_O_2_ treatment, the cells in 24-well plates were incubated with 10% neutral red solution to a final concentration of 0.033% at 37°C for 2 h. Thereafter, the culture supernatant was removed, the cells were fixed in 0.1% CaCl_2_, 0.5% formaldehyde, and the incorporated dye was solubilized in a 1% acetic acid, 50% ethanol solution. The absorbance was measured using a microplate reader with a 540 nm test wavelength and a 690 nm reference wavelength.

### 2.3. Generation of G6PD-Knockdown and Scrambled Control Hep G2 Cells

The cassette for expressing G6PD and scrambled control shRNA has been described elsewhere [14]. It was subcloned in pSUPER.retro. puro vector (Oligoengine, Seattle, WA, USA). The retroviral vectors were packaged into amphotropic virus using PT67 cells, as previously described [5, 15]. Hep G2 cells were transduced with the packaged virus and selected for stable transfectants in a medium containing 3 *μ*g/ml puromycin.

### 2.4. Western Blotting Analysis

Western blotting was performed as previously described [5, 15]. Briefly stated, the cells were rinsed with cold PBS, scraped, and collected by centrifugation. They were immediately lysed in lysis buffer (20 mM Tris-HCl (pH 8), 1% Triton X-100, 137 mM NaCl, 1.5 mM MgCl_2_, 10% glycerol, 1 mM EGTA, 1 mM NaF, 1 mM Na_3_VO_4_, 10 mM *β*-glycerophosphate, 1 mM phenylmethylsulfonyl fluoride, 1 *μ*g/ml leupeptin, and 1 *μ*g/ml aprotinin). Protein concentration of cell lysate was determined using the Bio-Rad protein assay kit (Bio-Rad Laboratories, Hercules, CA, USA). The sample was analyzed by SDS-PAGE and immunoblotting with anti-AMPK, anti-pAMPK, anti-NADK, and anti-actin antibodies.

### 2.5. Extraction of Cellular Metabolites

Extraction was performed as previously described with modifications [8, 16]. After removal of medium, cells were scraped in 80% methanol precooled at −80°C. The extract was collected, centrifuged at 14000 × g for 15 min, and extracted once more with 80% methanol. The extracts were pooled and completely dried under nitrogen gas. The sample was dissolved in 200 *μ*l 0.1% formic acid, centrifuged at 14000 × g for 15 min to remove debris, and subjected to LC-MS analysis.

### 2.6. LC-TOF-MS Analysis

Liquid chromatographic separation was achieved on a 100 mm × 2.1 mm ACQUITY 1.8 *μ*m HSST3 C18 column (Waters Corp.; Milford, MA, USA) using an ACQUITY TM Ultra Performance Liquid Chromatography (UPLC) system (Waters). The column was maintained at 40°C, and the flow rate was set at 0.5 ml/min. Sample was eluted from LC column using a linear gradient: 0–1.5 min, 1–25% B; 1.5–2.0 min, 25–98% B; 2.0–4.9 min, 98% B; and 5.0–7.0 min, 1% B (for reequilibration). Solvent A was 0.1% formic acid in water and solvent B was acetonitrile containing 0.1% formic acid. The lyophilized sample was dissolved with 200 *μ*l of water/acetonitrile (95 : 5, *v*/*v*). Mass spectrometry was performed on a Waters Q Tof-MS (SYNAPT G2S, Waters MS Technologies, Manchester, UK) operated in an ESI negative ion mode. The scan range was from 50 to 990 m/z. The desolvation gas flow was set to 1000 l/hr at 500°C, and temperature was set at 150°C. The capillary voltage and cone voltage were set at 2000 and 25 V for the ESI negative mode. Leucine encephalin, generating [M-H]^−^ ion (236.1035 m/z, 554.2615 m/z), was used as the lock mass at a concentration of 200 ng/ml and a flow rate of 10 *μ*l/min.

### 2.7. Data Processing

All data obtained in the negative ion mode were processed using Progenesis QI data analysis software (Nonlinear Dynamics, Newcastle, UK) for peak picking, alignment, and normalization to generate peak intensities for all features. The identities of features were obtained through search in METLIN (available at https://metlin.scripps.edu) [17] and Human Metabolome (available at http://www.hmdb.ca/) databases [18] and/or by comparison to both retention times and mass spectra of standard compounds. The MS data were analyzed by principal component analysis (PCA) and orthogonal partial least squares discriminate analysis (OPLS-DA) using SIMCA-P^+^ 13.0 (Umetrics, Sweden).

### 2.8. Metabolite Identification

For validation of the target metabolite, standards were analyzed under chromatographic conditions identical to that of the metabolite profiling experiment. MS and MS/MS analyses were performed under the same conditions as the metabolite profiling experiment. MS/MS spectra were collected at 0.3 second per scan, with a medium isolation window of ~4 m/z. The trap collision energy from 5 to 35 V was set.

## 3. Results

### 3.1. Distinct Impact of Hydrogen Peroxide on Global Metabolism of Hepatoma Cells

Our previous study has shown that GSH depletion significantly affects cellular metabolism and cell viability [8]. We sought to study if an oxidant can cause specific metabolic changes that are different from those brought about by GSH depletion. To test such possibility, we examined the changes in the metabolite profile of Hep G2 cells in response to H_2_O_2_ treatment. Hep G2 cells were treated without, or with 0.5 or 5 mM, H_2_O_2_ for a period ranging from 15 min to 2 hr. Cell extracts were analyzed using TOF-MS in negative ion mode. The experimental scheme is outlined in Supplemental Figure 1. The orthogonal partial least squares discriminate analysis (OPLS-DA) plot showed that the metabolite profile of cells treated with 5 mM H_2_O_2_ was significantly different from that of control cells. In contrast, there were mild changes in the metabolite profile of cells treated with 0.5 mM H_2_O_2_ versus that of control cells (Supplemental Figure 2B). Treatment of cells with 5 mM H_2_O_2_ for 4 hr resulted in considerable decline in viability, while treatment with 0.5 mM caused insignificant cell death (Supplemental Figure 2A).

**Figure 1:**
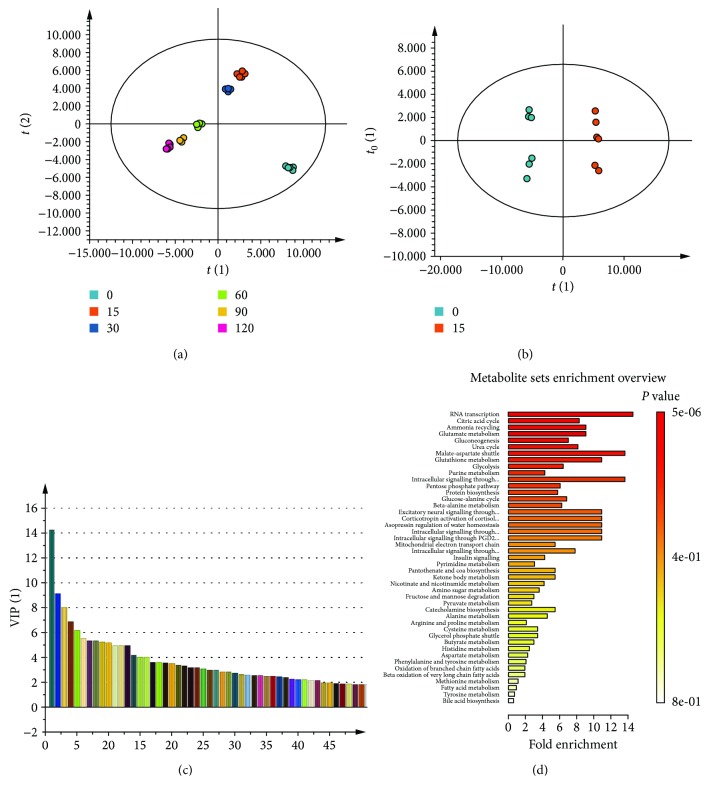
Early changes in metabolism of H_2_O_2_-treated hepatoma cells. Cells were treated with 5 mM H_2_O_2_ for 0, 15, 30, 60, 90, and 120 min and harvested for metabolomic analysis. Molecular features were identified by Progenesis QI, and the data were processed and analyzed using SIMCA-P. (a) Orthogonal partial least squares discriminant analysis (OPLS-DA) score plot for various treatment groups are shown. (b) OPLS-DA score plot of the 0 min- and 15 min-treated groups. The ellipse shown in the model (a, b) represents the Hotelling's *T* ^2^ with 95% confidence. (c) Variable importance in projection (VIP) plot of the OPLS-DA model for the 0 min- and 15 min-treated groups. Selected metabolites with a VIP value > 1.0 are presented. (d) The data were subjected to metabolite pathway analysis. A summary plot for metabolite set enrichment analysis (MSEA), in which metabolite sets are ranked according to the *p* value, is shown. The bar plot is color coded according to the calculated *p* values.

To study the early metabolic changes associated with H_2_O_2_-induced cell death, we focused on the analysis of datasets for 5 mM H_2_O_2_ treatment and control groups. As shown in OPLS-DA (Figure 1(a)), there were time-dependent changes in metabolites in H_2_O_2_-treated cells. Significant changes in metabolites occurred as early as 15 min after treatment (Figure 1(b)). Those features with variable importance in the projection (VIP) score greater than 1 correspond to differentially abundant metabolites and were selected for further analysis (Figure 1(c) and Supplemental Table 1). Enrichment analysis revealed changes in metabolites related to the metabolism of nucleotide, citric acid, glutamate, glucose, glutathione, and ammonia cycle in cells (Figure 1(d)). These findings suggest that these pathways may represent the early cellular metabolic response to H_2_O_2_-induced death.

### 3.2. H_2_O_2_-Induced Accumulation of S-1,7-BP and Fru-1,6-BP

**Figure 2:**
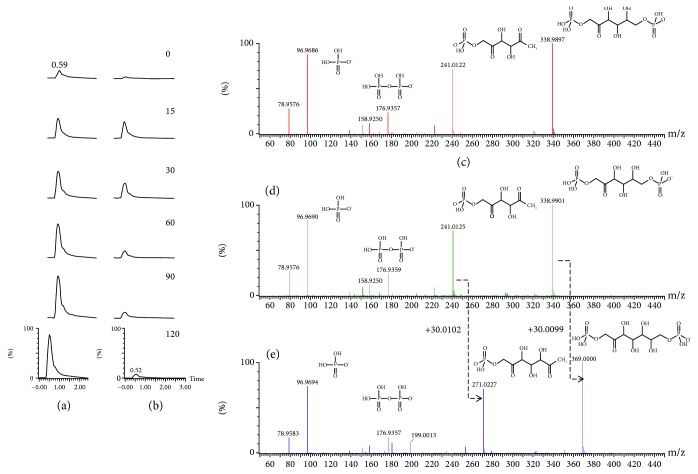
Oxidative stress-induced accumulation of Fru-1,6-BP and S-1,7-BP. The extracted ion chromatograms of Fru-1,6-BP (a) and S-1,7-BP (b) in Hep G2 cell treated with 5 mM H_2_O_2_ for 0, 15, 30, 60, 90, and 120 min are shown. Metabolite abundance is indicated by the peak intensity. A representative experiment is shown. The identities of Fru-1,6-BP (c, d) and S-1,7-BP (e) were validated by MS/MS analysis. The MS/MS spectrum of Fru-1,6-BP in cell specimen (d) was matched against that of standard (c). The spectrum of S-1,7-BP reveals a parent cation (369.0001 m/z) and a fragment ion (271.0227 m/z). The latter differs from the fragment ion of Fru-1,6-BP (241.0125 m/z) by a CHOH unit (30.01 m/z). For (c–e), the chemical structures of the fragment ions and their experimentally obtained m/z values are shown alongside the corresponding peaks.

Of the metabolites that changes in H_2_O_2_-treated cells, several metabolites related to the nonoxidative pentose phosphate pathway (PPP) are noteworthy. Fructose 1,6-bisphosphate (Fru-1,6-BP), the metabolite involved in regeneration of glucose, accumulated over time (Figure 2(a)). Intriguingly, the S-1,7-BP level rapidly increased by nearly 11.41-fold within 15 min of treatment, peaked at 30 min, and subsided (Figure 2(b); Supplemental Table 1). The identities of Fru-1,6-BP (Figure 2(c) & 2(d)) and S-1,7-BP (Figure 2(e)) were validated by MS/MS. Similarly, octulose-1,8-bisphosphate (O-1,8-BP) increased by roughly 6.75-fold at 15 min after treatment, remained elevated up to 60 min, and gradually declined afterwards (Supplemental Table 1). Previous studies have shown that S-1,7-BP and O-1,8-BP were present in hemolysate and thought to be synthesized by aldolase in a distinct type of nonoxidative PPP [19–21]. Our recent findings suggest that PPP is activated in response to treatment with high H_2_O_2_ concentration.

### 3.3. H_2_O_2_-Induced Changes in Redox and Energy Metabolism

GSH metabolism is affected by H_2_O_2_ treatment. The GSSG level increased to nearly 5 times the basal level within 30 min of treatment and stayed more or less steady throughout the treatment period. The decline in the GSH level was relatively modest during the same period (Supplemental Table 1). Such findings suggest that GSH buffer is largely maintained. However, other GSH-related intermediates were affected. For instance, the *S*-lactoylglutathione level increased over 130-fold within 90 min of treatment and declined afterwards (Supplemental Table 1).

Energy metabolism was drastically affected. Levels of nucleoside triphosphate, such as ATP and CTP, were drastically reduced, whereas monophosphate forms AMP, UMP, and GMP strongly increased in abundance. Moreover, cellular NAD^+^ decreased appreciably during H_2_O_2_ treatment (Supplemental Table 1). Besides, citrate accumulated over time during treatment. Apparently, cellular energy metabolism is adversely affected in the presence of high H_2_O_2_ concentration.

### 3.4. Knockdown of G6PD Impairs PPP and GSH Metabolism

**Figure 3:**
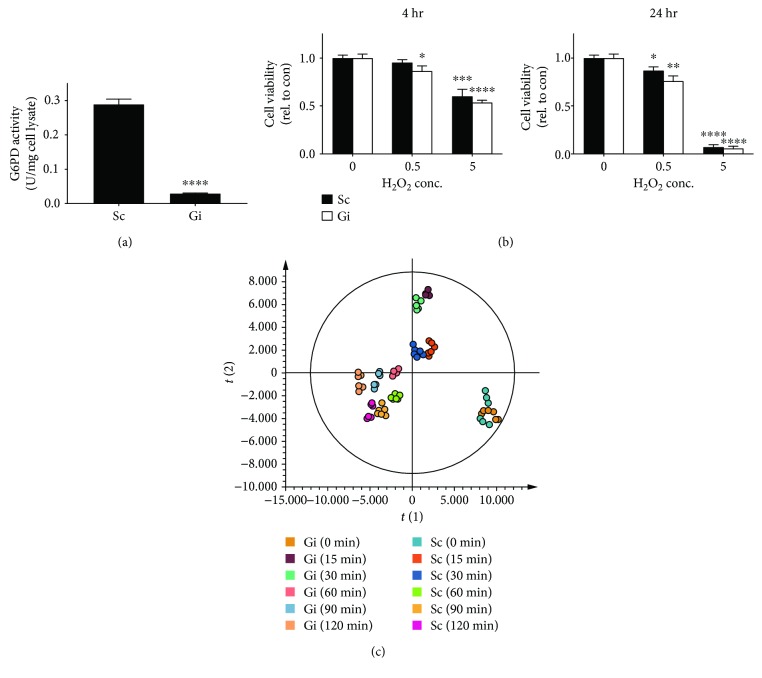
Temporal changes in metabolism in G6PD-deficient hepatoma cells. (a) G6PD activities in Sc and Gi cells were measured and are expressed in U/mg of cell lysate. Data are means ± SD, *n* = 6. (b) Sc and Gi cells were untreated or treated with 0.5 and 5 mM H_2_O_2_ for 4 and 24 hr, and their viabilities were determined. Data are means ± SD, *n* = 6. (c) Sc and Gi cells were treated with 5 mM H_2_O_2_ for 0, 15, 30, 60, 90, and 120 min and collected for metabolomic analysis. Data were analyzed as described in the legend of Figure 1. The OPLS-DA score plot of Sc and Gi cells treated for various times is shown.

**Figure 4:**
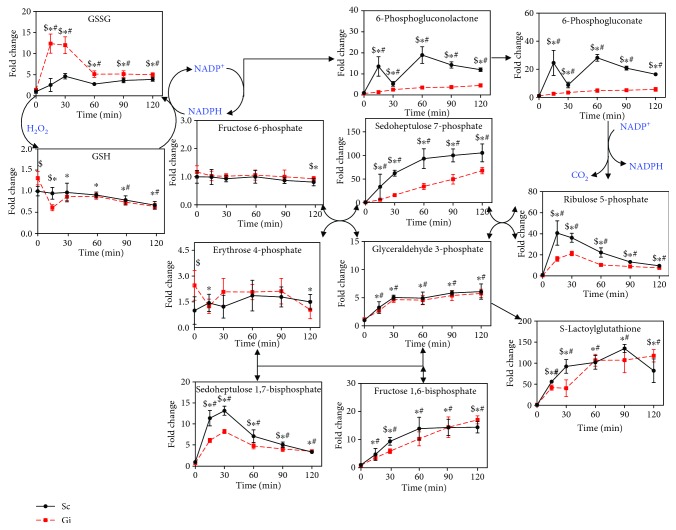
Changes in intermediates in PPP and glutathione metabolism. Analysis of metabolomic data that were obtained as described in the legend of Figure 3(c) reveals that a number of metabolites in PPP and glutathione metabolism change with time after H_2_O_2_ treatment. The levels of metabolites are expressed relative to that of untreated Sc cells. Data are means ± SD, *n* = 6. ^$^ *p* < 0.05, Sc cells vs Gi cells; ^∗^ *p* < 0.05, treated vs untreated Gi cells; and ^#^ *p* < 0.05, treated vs untreated Sc cells.

Diamide that induces mild changes in cellular GSH and GSSG in normal hepatoma cells does not significantly affect their viability [8]. In contrast, treatment with 5 mM H_2_O_2_, which caused similar changes in the abundance of GSH and GSSG as diamide, elicited cell death. It is wondered that redox parameters, such as GSSG/GSH redox potential, are not the sole determinant of the cellular outcome of H_2_O_2_ treatment. G6PD is critical to maintenance of NADPH and GSH. We studied the effect of G6PD knockdown on H_2_O_2_-induced changes in metabolism and cell physiology. We knocked down expression of *G6PD* gene in Hep G2 cells and derived knockdown (Gi) and control (Sc) cells. As expected, the G6PD activity in Gi cells was reduced by 90%, as compared with that in Sc cells (Figure 3(a)). H_2_O_2_ treatment induced a slightly higher degree of cell death in Gi cells than in Sc cells (Figure 3(b)). To examine the effect of H_2_O_2_ on metabolism, we treated Gi and Sc cells with 5 mM H_2_O_2_ for 15, 30, 60, 90, and 120 min and extracted them for metabolite profiling. Datasets were analyzed using multivariate statistical analyses. The OPLS-DA plots for Gi and Sc cells are shown in Figure 3(c). The metabolite profile of these cells changed in a time-dependent manner. As expected, PPP is adversely affected by G6PD deficiency (Figure 4). The levels of PPP intermediates, such as sedoheptulose 7-phosphate (S7P), 6-phosphoglucono-*δ*-lactone, 6-phosphogluconate (6PG), and ribulose 5-phosphate, in Gi cells were significantly lower than those in Sc cells. Additionally, the S-1,7-BP level was largely lowered in Gi cells, advocating that S-1,7-BP is an intermediate formed in the nonoxidative branch of PPP.

Temporal changes in GSH and GSSG were also followed (Figure 4). The levels of GSSG in Gi and Sc cells increased within 30 min of treatment and were significantly lowered at 60 min after treatment. The extent of the surge in the GSSG level was much higher in the former than in the latter. The basal GSH level in Gi cells was higher than that in Sc cells. Upon H_2_O_2_ treatment, the GSH level in Gi cells showed a transient large drop and gradually stabilized at a value lower than the basal level. Meanwhile, the GSH abundance in Sc cells declined gradually with time and was not significantly different from that in Gi cells at 120 min. Apparently, the dynamic changes in the GSSG and GSH pools in these cells, particularly Gi cells, represent their effort to restore GSH homeostasis. Notwithstanding the differences in the kinetics and extent of changes in GSH and GSSG levels between Gi and Sc cells, H_2_O_2_ treatment results in the same outcome—cell death. These findings, together with our previous studies on diamide, indicate that changes in GSH and GSSG levels per se do not determine the fate of cells exposed to high concentration of H_2_O_2_. It is likely that events consequent to changes in redox homeostasis may be linked to initiation of H_2_O_2_-induced cell death.

### 3.5. H_2_O_2_ Treatment Causes Anomalous Energy Metabolism

**Figure 5:**
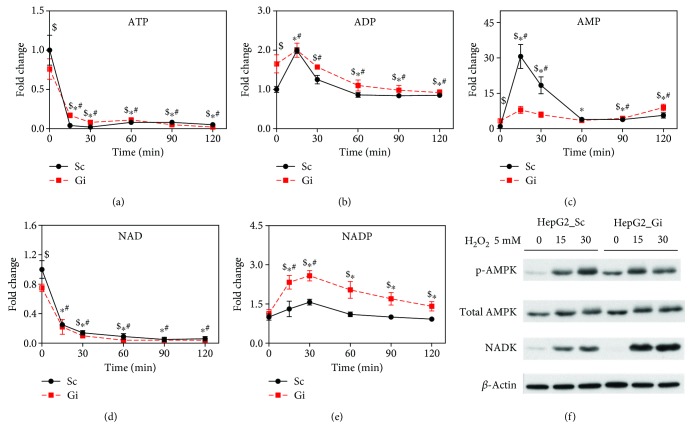
Oxidative stress-induced changes in energy and redox metabolism. Sc and Gi cells were treated with 5 mM H_2_O_2_ for 0, 15, 30, 60, 90, and 120 min. The cells were harvested, and cellular levels of ATP (a), ADP (b), AMP (c), NAD^+^ (d), and NADP^+^ (e) were determined by LC-MS. The levels of metabolites are expressed relative to those of untreated Sc cells. Data are means ± SD, *n* = 6. (f) Sc and Gi cells were untreated or treated with 5 mM H_2_O_2_ for 0, 15, and 30 min and harvested for immunoblotting with antibodies to p-AMPK, AMPK, NADK, and actin. A representative experiment out of three is shown. ^$^ *p* < 0.05, Sc cells vs Gi cells; ^∗^ *p* < 0.05, treated vs untreated Gi cells; and ^#^ *p* < 0.05, treated vs untreated Sc cells.

**Figure 6:**
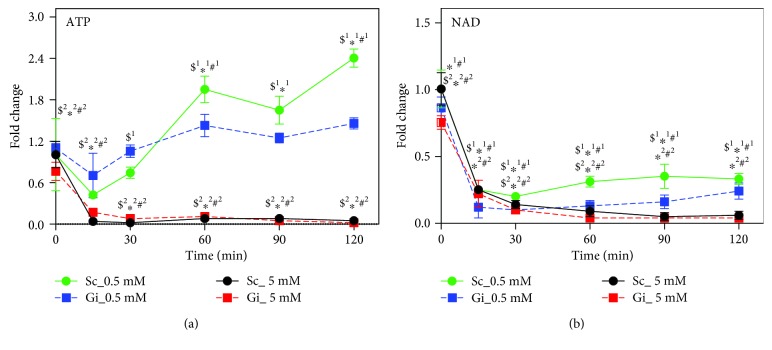
ATP and NAD exhaustion accounts for death of hepatoma cells subject to oxidative stress. Sc and Gi cells were treated with 0.5 or 5 mM H_2_O_2_ for 0, 15, 30, 60, 90, and 120 min and were extracted for LC-MS analysis of ATP (a) and NAD^+^ (b). The levels of metabolites are expressed relative to that of untreated Sc cells. Data are means ± SD, *n* = 6. ^$1^ *p* < 0.05 or ^$2^ *p* < 0.05, Sc cells vs Gi cells treated with 0.5 mM or 5 mM H_2_O_2_; ^∗1^ *p* < 0.05 or ^∗2^ *p* < 0.05, treated vs untreated Gi cells upon treatment with 0.5 mM or 5 mM H_2_O_2_; and ^#1^ *p* < 0.05 or ^#2^ *p* < 0.05, treated vs untreated Sc cells upon treatment with 0.5 mM or 5 mM H_2_O_2_.

Our previous study has shown that energy metabolism is hampered in diamide-treated cells [8]. As shown in Supplemental Table 1, ATP was significantly reduced in H_2_O_2_-treated cells. We proceeded to examine the changes in levels of ATP in Gi and Sc cells receiving H_2_O_2_ treatment. ATP levels in Gi and Sc cells declined dramatically with treatment time (Figure 5(a)). Basal ADP and AMP levels in Sc were substantially lower than those in Gi cells. The ADP levels of Sc and Gi cells increased, peaked at 15 min, and declined thereafter (Figure 5(b)). The AMP levels of Sc and Gi cells spiked at 15 min and returned to values higher than their basal levels (Figure 5(c)). Accumulation of AMP was associated with phosphorylation of AMPK at Thr172 (Figure 5(f)). These findings suggest that H_2_O_2_ induces ATP depletion. As treatment of Sc and Gi cells with 0.5 mM H_2_O_2_ did not cause their death, we examined the effect of 0.5 mM H_2_O_2_ on the cellular ATP content. As shown in Figure 6(a), in contrast to 5 mM H_2_O_2_, 0.5 mM H_2_O_2_ induced a significant increase in the ATP level over time.

### 3.6. H_2_O_2_ Treatment Induces Expression of NADK and Biosynthesis of NADP^+^

The transient increase in GSSG in Gi cells prompts us to study if pathways other than PPP provide reducing equivalents for its reduction. It is probable that NADP^+^ synthesis is upregulated during H_2_O_2_ treatment. Consistent with this, the NADP^+^ level in Gi and Sc cells increased substantially within 30 min of treatment. The magnitude of the increase was significantly greater in the former than in the latter (Figure 5(e)). The basal NAD^+^ level in Gi cells was lower than that in Sc cells. Upon H_2_O_2_ treatment, it decreased drastically (Figure 5(d)).

NADK, which catalyzes NAD^+^ phosphorylation and its conversion to NADP^+^, may be part of the compensatory mechanism. To study whether NADK is involved, we examined NADK expression in H_2_O_2_-treated Sc and Gi cells. As shown in Figure 5(f), expression of NADK increased in both cells, with the induction fold being higher in Gi cells than in Sc cells.

### 3.7. Inhibition of PARP-Mediated NAD^+^ Depletion Partially Protects Cells from Death

H_2_O_2_ treatment causes NAD^+^ depletion. There were large drops in the NAD^+^ level in Gi and Sc cells within 15 min of treatment with either 0.5 or 5 mM H_2_O_2_ (Figure 6(b)). For the 5 mM treatment group, their NAD^+^ levels continued to decline from 15 min onward. In contrast, for the 0.5 mM treatment group, the NAD^+^ levels of Gi and Sc cells stabilized at 15 min posttreatment and gradually rebounced to values that were about 30% of their respective basal levels.

**Figure 7:**
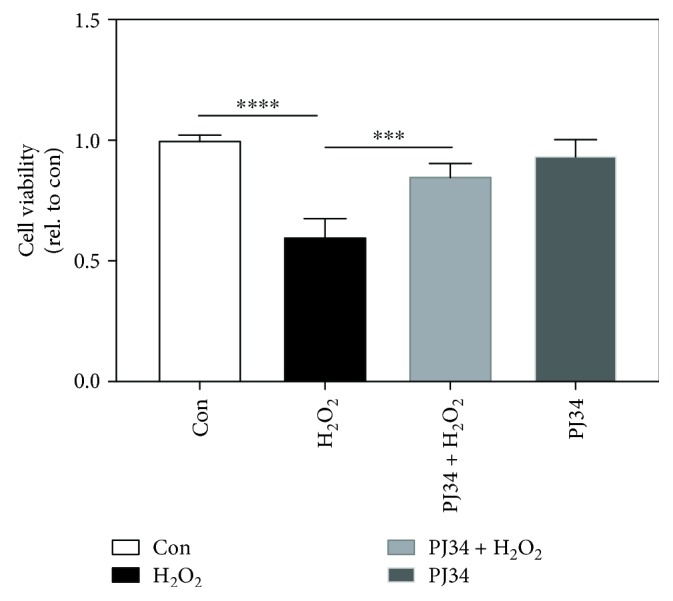
Inhibition of PARP partially rescues hepatoma cells from death. Hep G2 cells were pretreated with 30 *μ*M PJ34 for 2 hr prior to treatment with 5 mM H_2_O_2_. Cell viability was determined and is expressed relative to control. Data are means ± SD, *n* = 6. ^∗∗∗^ *p* < 0.005 and ^∗∗∗∗^ *p* < 0.001.

One of the routes, through which NAD^+^ is rapidly utilized and exhausted, is poly (ADP-ribose) polymerase (PARP). ROS are known to cause single-strand breaks and PARP activation [22]. To test if PAPR-mediated depletion of NAD^+^ accounts for cell death, we cotreated Hep G2 cells with PARP inhibitor PJ34 and H_2_O_2_ and examined their viability. Treatment of Hep G2 cells with PJ34 enhanced viability but did not completely block their death (Figure 7). These findings suggest that PARP-mediated NAD^+^ depletion contributes partly to the death process.

## 4. Discussion

In this study, we have applied the metabolomic approach for studying the metabolic response of hepatoma cells to H_2_O_2_. High concentration of H_2_O_2_ significantly alters the fluxes of metabolic pathways. Some of these pathways, such as the PPP and NADK pathway, are activated to restore redox homeostasis. Despite their effectiveness, these pathways lead to excessive consumption of NAD^+^ and ATP. Our findings support the notion that impairment of energy metabolism precipitates cell death.

H_2_O_2_-induced changes in metabolism are different from what we observed in diamide-treated cells. Diamide reacts with thiols and causes GSH depletion. It induces time-dependent changes in metabolic pathways, such as GSH biosynthesis, amino acid metabolism, and energy metabolism, in hepatoma cells [8]. GSH biosynthesis and amino acid uptake are not enhanced in H_2_O_2_-treated cells. The disparity may lie in the fact that diamide is highly effective in the conversion of GSH to GSSG and hence GSH pool diminution. This may lead to the relief of the inhibitory effect of GSH on glutamate cysteine ligase (GCL) [23] and to the subsequent *γ*-glutamylcysteine formation. In agreement with this, diamide is superior to H_2_O_2_ in its ability to deplete the cellular GSH pool [24]. Nonetheless, there are metabolic changes commonly induced by H_2_O_2_ and diamide. For instance, energy metabolism is adversely affected [8]. PPP is important to the provision of reducing equivalents [7].

PPP is activated in response to oxidant treatment. Levels of intermediates of normal PPP, such as sedoheptulose 1,7-bisphosphate and ribulose 5-phosphate, increased with treatment time. We detected metabolites that are supposedly to be formed in the L-type pathway [25]. These metabolites include S-1,7-BP and O-1,8-BP. S-1,7-BP has been found in erythrocytes and liver tissues. It is conjectured that S-1,7-BP can be formed from dihydroxyacetone phosphate and erythrose-4-phosphate (E4P) via aldolase activity. E4P can replace glyceraldehye-3-phosphate (G3P) as a substrate for S-1,7-BP synthesis in extracts of mammalian tissues such as the liver and muscle [21, 26]. Additionally, it may be formed from fructose 1,6-bisphosphate and E4P. Alternatively, 6-phosphofructokinase may catalyze S-1,7-BP formation from sedoheptulose 7-phosphate. O-1,8-BP may arise from condensation of DHAP and ribose 5-phosphate [27]. In our present study, we validate that these metabolites are formed in nonoxidative PPP. Knockdown of the *G6PD* gene significantly reduced the levels of these metabolites. In this sense, S-1,7-BP is considered a marker of increased PPP activity.

Our previous study has shown that diamide treatment caused about 11% decrease in the GSH pool in hepatoma cells at 60 min posttreatment [8]. In the present study, H_2_O_2_ treatment resulted in around a 10% decrease in this pool. Despite the similarity in the extent of change, H_2_O_2_ induced cell death, while diamide did not. Such findings suggest that factors other than simply a change in GSH parameter contribute to cell death.

H_2_O_2_ treatment causes anomalous energy production. The ATP level decreased continually throughout treatment and became virtually exhausted. ADP and AMP levels transiently rose and gradually subsided. The increases in ADP and AMP levels activate AMPK and cause influx of glucose into the glycolytic pathway. The increase in glycolytic flux is indicated by increases in levels of fructose 1,6-bisphosphate and glyceraldehyde 3-phosphate. Additionally, such increases may promote the nonoxidative branch of PPP for glucose regeneration and continual operation of PPP. Furthermore, accumulation of glyceraldehyde 3-phosphate is accompanied by formation of lactoylglutathione. Lactoylglutathione is derived from methylglyoxal and glutathione via the activity of glyoxalase I [28, 29]. This represents a pathway involved in detoxification of methylglyoxal, which is able to glycate nucleic acid and protein and conduces to advanced glycation end product (AGE) formation [30]. Methylglyoxal is formed either nonenzymatically from dihydroxyacetone phosphate and glyceraldehyde 3-phosphate or enzymatically via a triosephosphate isomerase-mediated process [28]. It has been demonstrated that a low NAD^+^ level promotes generation of methylglyoxal [31]. H_2_O_2_-induced deficit in NAD^+^ probably reduces the flux of glyceraldehyde 3-phospahte-catalyzed reaction and makes it favorable for diversion of triose phosphate toward methylglyoxal formation. An additional point is noteworthy. The total pool of adenine nucleotides dwindles, suggesting their transformation to other molecules. We observed that the level of diadenosine diphosphate spiked within the first 30 min of H_2_O_2_ treatment and remained elevated. Diadenosine diphosphate (Ap_2_A) is formed in a side reaction catalyzed by aminoacyl tRNA synthetase in the absence of cognate tRNA [32]. Diadenosine oligophosphate is proposed as a novel class of signaling molecules. For instance, Ap_3_A binds to the Fhit protein and, as a complex, serves as effector molecule with tumor suppressor activity [33]. Ap_n_A interacts with P2Y and P2X receptors and elicits downstream signaling [34]. Ap_2_A protects neutrophils from apoptosis [35]. In addition, Ap_2_A stimulates growth of vascular smooth muscle cells [36]. It is probable that Ap_2_A might be generated in H_2_O_2_-treated cells in an attempt to rescue them from demise. Moreover, another metabolite adenylosuccinate increased in abundance within 15 min of treatment and gradually declined. It is an intermediate formed in the path of conversion of IMP to AMP. H_2_O_2_-induced change in the adenylosuccinate pool probably represents a biochemical response of cells in the face of ATP depletion. These cells upregulate purine biosynthesis to make up for the deficit.

AMPK is composed of 3 subunits, including catalytic *α* subunit and regulatory *β* and *γ* subunits. It acts as an important energy sensor molecule that controls cellular physiology [37, 38]. AMPK is activated by an increase in the AMP/ATP ratio under the stressful condition that hinders ATP generation and accelerates its consumption [39]. Its activity can also be modulated by ADP and NAD^+^ [40–42]. Moreover, AMPK can be regulated indirectly through reactive nitrogen species-mediated activation of upstream kinases LKB1 and CaMKKb or through S-glutathionylation or S-nitrosylation of cysteine residues of the AMPK *α* subunit [43, 44]. H_2_O_2_-induced increases in AMP and ADP, and probably oxidative modification, activate AMPK. AMPK activation enhances hepatic glucose uptake via Glut2 [45]. AMPK is able to phosphorylate phosphofructokinase 2 [46], which generates fructose 2,6-bisphosphate, a potent activator of phosphofructokinase 1. Increase in fructose 1,6-bisphosphate in H_2_O_2_-treated cells may be accounted for by an increased flux of phosphofructokinase 1-catalyzed reaction.

H_2_O_2_-induced changes in nicotinamide adenine nucleotide pools have important implication in biochemical and cellular processes. NAD^+^ depletion, amid the maintenance of the NADP^+^ pool, is associated with increased NADK expression. Menadione increases NADK activity and causes NAD^+^ depletion in colon epithelial cells [47]. NADK activity increases in diamide-treated G6PD-deficient cells [8]. It is plausible that upregulation of NADK represents a mechanism for maintenance of the NADP^+^ pool [48, 49]. Mammalian NADK catalyzes formation of NADP^+^ at the expense of NAD^+^ and ATP. Increased NADK activity in H_2_O_2_-treated cells, particularly Gi cells, contributes to diminution of the NAD^+^ and ATP pools. Oxidative damage-induced DNA damage leads to activation of poly (ADP ribose) polymerase (PARP) and NAD^+^ depletion [50, 51]. Treatment with the PARP inhibitor partially protects H_2_O_2_-treated cells from death, implying that activation of PARP is involved in cell death. These findings suggest that depletion of NAD^+^ and ATP can trigger cell death. Deficit in NAD^+^ leads to reduction in glycolysis and oxidative phosphorylation and hence to insufficient ATP production. When NAD^+^ and ATP levels fall below certain thresholds, cells are destined to die.

Accumulation of citrate in H_2_O_2_-treated cells is interesting. H_2_O_2_ increases the intracellular glutamine level and concomitantly reduces the glutamate level. Glutamine is available in culture medium as energy source and is converted to glutamate by glutaminase. Glutamate can be catabolized to *α*-ketoglutarate via action of glutamate dehydrogenase. *α*-Ketoglutarate is metabolized via forward reactions of the TCA cycle or transformed via isocitrate dehydrogenase and aconitase to citrate in a process known as reductive carboxylation. For the former process, citrate abundance is elevated as a consequence of reduced mitochondrial aconitase activity. Aconitase is uniquely sensitive to superoxide-mediated inactivation [52, 53]. Another nonexclusive possibility is that reductive carboxylation is essential to the maintenance of redox balance [54] and is probably upregulated as a response to oxidative stress. Citrate formed by reductive carboxylation is transported to the cytoplasm, where it is acted upon by cytosolic aconitase and isocitrate dehydrogenase to generate NADPH. H_2_O_2_-induced citrate accumulation may represent such an antioxidative mechanism.

A number of mechanisms come into play to maintain redox homeostasis. Activation of PPP protects cells from oxidative damage within the first few minutes of treatment [6]. Reductive carboxylation and NADK may provide an additional supportive role for NADPH generation. PARP activation is involved in signaling of the DNA repair process. These antioxidative and repair mechanisms may be activated in cells subject to oxidative stress and consume NAD^+^ and ATP. In milieu of strong oxidative stress, excessive utilization of these key metabolites leads to cellular energy stress and cell death.

## Data Availability

The metabolic and oxidative data used to support the findings of this study are available from the corresponding author upon request.

## Supplementary Materials

Supplementary Materials Supplemental Table 1: a list of metabolites differentially abundant in the 0 min- versus 15 min-treated groups. Supplemental Figure 1: the workflow of our experiment. Supplemental Figure 2: temporal changes in metabolism in H_2_O_2_-treated Hep G2 cells.
