# Supplementary material for: Sedoheptulose-1,7-bisphospate Accumulation and Metabolic Anomalies in Hepatoma Cells Exposed to Oxidative Stress
Source: Oxid Med Cell Longev. 2019 Jan 13;2019:5913635. doi: 10.1155/2019/5913635 (PMC6348915; doi:10.1155/2019/5913635)
Supplement: Supplementary Materials — Supplemental Table 1: a list of metabolites differentially abundant in the 0 min- versus 15 min-treated groups. Supplemental Figure 1: the workflow of our experiment. Supplemental Figure 2: temporal changes in metabolism in H2O2-treated Hep G2 cells. [file 5913635.f1.docx]

**Supplementary Materials**

**Supplemental Table 1**. A list of metabolites differentially abundant in 0 min- versus 15 min-treatment groups

| **Metabolites** | **Adducts** | **m/z** | **RT** | **VIP** | **0 min** | **15 min** | **30 min** | **60 min** | **90 min** | **120 min** |
| --- | --- | --- | --- | --- | --- | --- | --- | --- | --- | --- |
| ATP | M-H | 505.9869 | 0.59 | 14.25 | 1.00 | 0.04 | 0.02 | 0.08 | 0.08 | 0.05 |
| ADP | M-H | 426.0215 | 0.59 | 9.15 | 1.00 | 1.99 | 1.25 | 0.86 | 0.84 | 0.85 |
| UMP | M-H | 323.0282 | 0.66 | 8.06 | 1.00 | 14.47 | 8.76 | 3.94 | 4.14 | 4.85 |
| AMP | M-H | 346.0550 | 0.70 | 6.88 | 1.00 | 30.64 | 18.38 | 3.89 | 3.88 | 5.62 |
| Citric acid | M-H | 191.0194 | 0.70 | 6.21 | 1.00 | 3.08 | 3.79 | 5.74 | 6.78 | 7.23 |
| Adenosine diphosphate ribose | M-H | 558.8988 | 0.81 | 5.56 | 1.00 | 0.27 | 0.23 | 0.36 | 0.29 | 0.29 |
| Sedoheptulose 1,7-bisphosphate | M-H | 368.9984 | 0.60 | 5.26 | 1.00 | 11.41 | 13.18 | 7.14 | 5.11 | 3.38 |
| L-Glutamic acid | M-H | 146.0457 | 0.49 | 5.21 | 1.00 | 0.61 | 0.50 | 0.31 | 0.29 | 0.25 |
| Ribulose 5-phosphate | M-H | 229.0108 | 0.48 | 4.97 | 1.00 | 40.7 | 36.21 | 22.25 | 13.27 | 9.58 |
| UDP-*N*-acetylglucosamine | M-H | 606.0737 | 0.53 | 3.68 | 1.00 | 1.09 | 1.15 | 0.98 | 0.81 | 0.68 |
| CTP | M-H | 481.9758 | 0.62 | 3.65 | 1.00 | 0.21 | 0.10 | 0.30 | 0.34 | 0.27 |
| Oxidized glutathione | M-H | 611.1442 | 0.70 | 3.60 | 1.00 | 2.57 | 4.64 | 2.79 | 3.64 | 3.89 |
| UDP | M-H | 402.9943 | 0.52 | 3.58 | 1.00 | 1.23 | 0.97 | 0.81 | 0.80 | 0.64 |
| *S*-Lactoylglutathione | M-H | 378.0966 | 1.11 | 3.22 | 1.00 | 56.14 | 92.28 | 101.88 | 134.91 | 82.15 |
| GDP | M-H | 442.0159 | 0.63 | 3.15 | 1.00 | 1.93 | 1.04 | 1.24 | 1.70 | 1.84 |
| GMP | M-H | 362.0496 | 0.70 | 3.05 | 1.00 | 26.17 | 14.55 | 4.28 | 7.02 | 12.05 |
| *N*-acetyl-aspartate | M-H | 174.0400 | 0.70 | 2.29 | 1.00 | 0.55 | 0.61 | 0.35 | 0.31 | 0.28 |
| L-Tyrosine | M-H | 180.0661 | 1.04 | 1.99 | 1.00 | 0.82 | 0.80 | 0.91 | 0.94 | 0.89 |
| Fructose 1,6-bisphosphate | M-H | 338.9881 | 0.48 | 1.95 | 1.00 | 4.74 | 9.41 | 13.92 | 14.36 | 14.4 |
| L-Aspartic acid | M-H | 132.0296 | 0.47 | 1.88 | 1.00 | 2.49 | 3.35 | 3.59 | 3.99 | 4.21 |
| Adenylsuccinic acid | M-H | 462.0650 | 1.10 | 1.61 | 1.00 | 121.01 | 82.09 | 17.07 | 24.04 | 46.24 |
| CMP | M-H | 322.0438 | 0.59 | 1.59 | 1.00 | 1.60 | 1.03 | 0.65 | 0.78 | 0.89 |
| NADP | M-H | 742.0674 | 0.67 | 1.57 | 1.00 | 1.31 | 1.57 | 1.10 | 1.00 | 0.92 |
| Coenzyme A | [M-2H] | 382.5489 | 1.23 | 1.39 | 1.00 | 0.25 | 0.11 | 0.36 | 0.46 | 0.28 |
| Malic acid | M-H | 133.0137 | 0.65 | 1.39 | 1.00 | 0.72 | 0.85 | 1.21 | 1.48 | 1.42 |
| Diadenosine diphosphate | M-H | 675.1186 | 0.70 | 1.33 | 1.00 | 128.32 | 79.49 | 21.06 | 25.79 | 38.14 |
| NAD | M-H | 662.0996 | 0.71 | 1.31 | 1.00 | 0.25 | 0.14 | 0.09 | 0.05 | 0.06 |
| Octulose-1,8-bisphosphate | M-H | 399.0089 | 0.48 | 1.28 | 1.00 | 6.75 | 11.18 | 10.04 | 7.81 | 5.96 |
| Glutathione | M-H | 306.0762 | 0.70 | 1.26 | 1.00 | 0.95 | 0.97 | 0.91 | 0.79 | 0.67 |
| Pantothenic acid | M-H | 218.1031 | 1.40 | 1.19 | 1.00 | 0.92 | 1.00 | 0.99 | 0.98 | 0.80 |
| L-Glutamine | M-H | 145.0613 | 0.48 | 1.15 | 1.00 | 1.30 | 1.18 | 1.31 | 1.25 | 1.19 |

The metabolites were differentially abundant in 0 min- versus 15 min-treatment groups and selected according to criteria (VIP >1.0 & fold change>2).

**Supplemental Figure Legends**

Supplemental Figure 1**.** The workflow of our experiment. Cells were treated with H_2_O_2_ for different periods, and processed for untargeted metabolomic analysis. The data were analyzed using SIMCA-P. Metabolites were identified through database search and/or comparison to spectra of reference compounds.

Supplemental Figure 2. Temporal changes in metabolism in H_2_O_2_-treated Hep G2 cells. (A) Hep G2 cells were un- or treated with 0.5 and 5 mM H_2_O_2_ for 2 and 4 hr, and their viabilities were determined. Data are means±SD, n=6. ***p<0.005, 2- vs. 4-hr treatment groups. (B) Hep G2 cells were treated with 0.5 or 5 mM H_2_O_2_ for 0, 15, 30, 60, 90 and 120 min, and collected for metabolomic analysis. Data were analyzed as described in the legend of Fig. 1. The OPLS-DA score plot of Hep G2 cells treated for various times is shown.

Supplemental Figure 1. The workflow of our experiment.


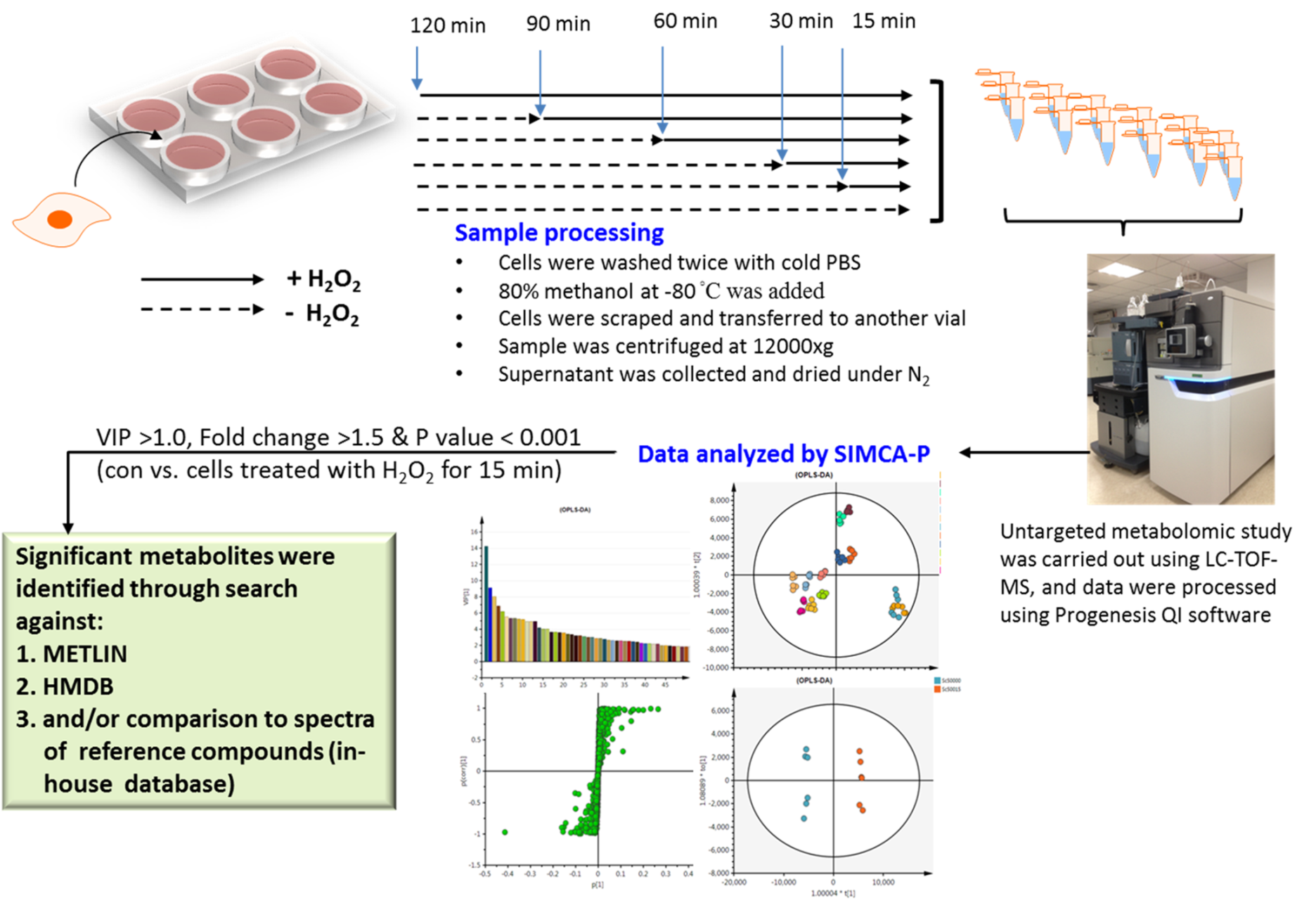


Supplemental Figure 2. Temporal changes in metabolism in H_2_O_2_-treated Hep G2 cells.

**
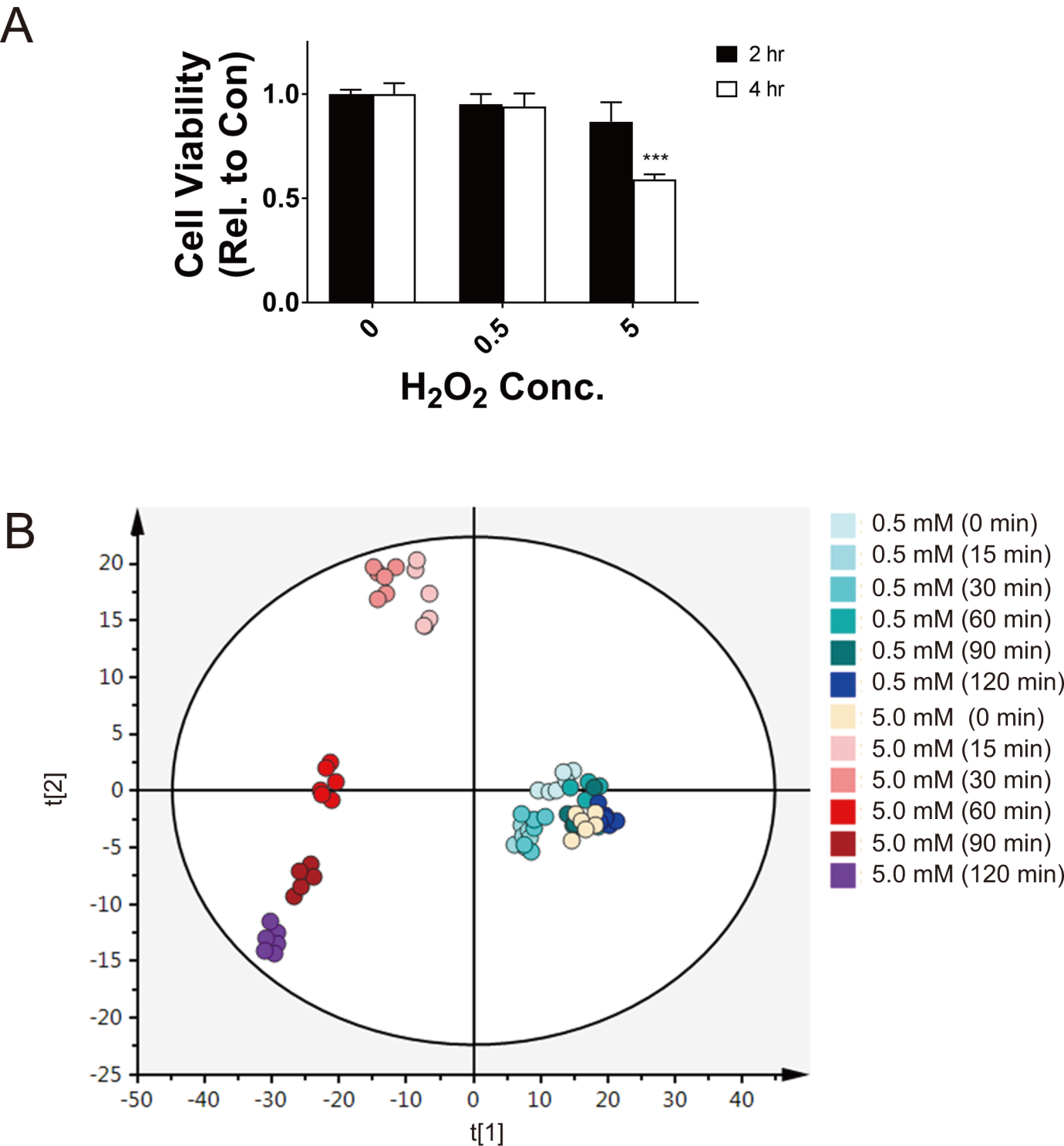
**
